# Analysis of Optimal Health-Related Quality of Life Measures in Patients Waitlisted for Lung Transplantation

**DOI:** 10.1155/2020/4912920

**Published:** 2020-03-05

**Authors:** Junko Tokuno, Toyofumi F Chen-Yoshikawa, Toru Oga, Takahiro Oto, Tomoyo Okawa, Yoshinori Okada, Miki Akiba, Masaki Ikeda, Daisuke Nakajima, Masatsugu Hamaji, Hideki Motoyama, Akihiro Aoyama, Maki Isomi, Kazuo Chin, Hiroshi Date

**Affiliations:** ^1^Department of Thoracic Surgery, Graduate School of Medicine, Kyoto University, Kyoto, Japan; ^2^Department of Thoracic Surgery, Graduate School of Medicine, Nagoya University, Nagoya, Japan; ^3^Department of Respiratory Medicine, Kawasaki Medical School, Kurashiki, Japan; ^4^Organ Transplant Center, Okayama University Hospital, Okayama, Japan; ^5^Department of Thoracic Surgery, Institute of Development, Aging and Cancer, Tohoku University, Sendai, Japan; ^6^Division of Organ Transplantation, Tohoku University Hospital, Sendai, Japan; ^7^Department of Respiratory Care and Sleep Control Medicine, Graduate School of Medicine, Kyoto University, Kyoto, Japan

## Abstract

**Background:**

Improving health-related quality of life (HRQL) is an important goal of lung transplantation, and St. George's Respiratory Questionnaire (SGRQ) is frequently used for assessing HRQL in patients waitlisted for lung transplantation. We hypothesized that chronic respiratory failure (CRF)-specific HRQL measures would be more suitable than the SGRQ, considering the underlying disease and its severity in these patients.

**Methods:**

We prospectively collected physiological and patient-reported data (HRQL, dyspnea, and psychological status) of 199 patients newly registered in the waiting list of lung transplantation. CRF-specific HRQL measures of the Maugeri Respiratory Failure Questionnaire (MRF) and Severe Respiratory Insufficiency Questionnaire (SRI) were assessed in addition to the SGRQ.

**Results:**

Compared to the MRF-26 and SRI, the score distribution of the SGRQ was skewed toward the worse ends of the scale. All domains of the MRF-26 and SRI were significantly correlated with the SGRQ. Multiple regression analyses to investigate factors predicting each HRQL score indicated that dyspnea and psychological status accounted for 12% to 28% of the variance more significantly than physiological measures did. The MRF-26 Total and SRI Summary significantly worsened from the baseline to 1 year (*p* < 0.001 and *p* < 0.001 and *p* < 0.001 and

**Conclusions:**

The MRF-26 and SRI are valid, discriminative, and responsive in patients waitlisted for lung transplantation. In terms of the score distribution and responsiveness, CRF-specific measures may function better in their HRQL assessment than the currently used measures do.

## 1. Introduction

Improvements of survival and functional outcomes have been the two major aims of lung transplantation since it was established as an optional treatment for patients suffering from end-stage lung diseases. With the developments in transplant management, the three- and five-year survival rates after double lung transplantation have improved to 69% and 59%, respectively [1]. Under these circumstances, improving the health-related quality of life (HRQL) of the patients is an important goal owing to the physical, psychological, and social limitations in daily life afforded by severe lung diseases.

Although previous studies have indicated the improvements in HRQL after lung transplantation, the most appropriate questionnaire to assess HRQL for patients waitlisted for lung transplantation remains uncertain. The Medical Outcomes Study 36-item short form (SF-36) [2] and St. George's Respiratory Questionnaire (SGRQ) [3] are the two most frequently used measurements in this regard [4, 5]. As the SF-36 is a generic HRQL questionnaire and is not specific to particular lung diseases, a disease-specific HRQL questionnaire is preferred to adequately detect changes in HRQL following specific interventions [6]. The SGRQ is a respiratory-specific disease questionnaire and is preferred in clinical trials owing to its better responsiveness. However, it was originally established for patients with mild to severe chronic airway diseases such as chronic obstructive pulmonary disease (COPD) and asthma, and very severe patients with chronic respiratory failure (CRF) were not accounted for in its validation [3]. Therefore, it may not be suitable for patients waitlisted for lung transplantation, who mostly tend to present CRF owing to various underlying end-stage lung diseases that are not limited to COPD and asthma.

The two CRF-specific HRQL measurements [7] are the Maugeri Respiratory Failure Questionnaire (MRF) [8] and the Severe Respiratory Insufficiency Questionnaire (SRI) [9, 10]. While the MRF is valid in patients using noninvasive ventilation (NIV) and/or long-term oxygen therapy (LTOT), it was first validated for COPD or kyphoscoliosis [7]. On the other hand, SRI was specifically validated as an instrument for patients receiving long-term NIV for various disorders such as COPD, restrictive thoracic disorders, neuromuscular disorders, and obesity hypoventilation syndrome [7]. We hypothesized that the MRF and SRI would be more suitable than the currently used measurements in evaluating HRQL for lung transplant candidates. Then, we assessed the HRQL of patients waitlisted for lung transplantation in Japan, using the MRF and SRI in addition to the SGRQ.

## 2. Methods

### 2.1. Patients

We prospectively recruited 199 patients who were newly registered to a waitlist of lung transplantation between 2009 and 2015. The study was approved by the institutional ethical boards of three centers, namely, Kyoto University Hospital, Tohoku University Hospital, and Okayama University Hospital. All patients provided written informed consent. Patients aged under 17 years and those who had previously undergone heart and lung transplantation were excluded. We assessed patient age, body mass index (BMI), pulmonary function, arterial blood gas, and patient-reported measurements of HRQL, dyspnea, and psychological status at the timing of registration to the waitlist. With regard to pulmonary function, forced expiratory volume in one second (FEV_1_) and forced vital capacity (FVC) were measured. In addition, stable patients underwent the 6-minute walk test, and the distance (6MWD) was recorded.

### 2.2. Patient-Reported Measurements

HRQL was evaluated using the Japanese versions of the SGRQ [11], MRF-26 [12], and SRI [12–14]. The SGRQ was originally developed for patients with chronic airflow limitations such as COPD or asthma [3]. However, it has been used in various respiratory diseases including pulmonary fibrosis, bronchiectasis, lymphangioleiomyomatosis (LAM), sarcoidosis, pulmonary hypertension, and bronchiolitis obliterans. The SGRQ has three components: Symptoms, Activities, and Impacts, and the total score was calculated from the summary of these three components. The MRF-26 is a modified version of the original MRF-28 designed for patients with CRF [8, 15]. It has two domains: daily activities and perceived disability, and the total score was calculated from the summary of the two domains. The SRI was originally validated for patients with CRF receiving long-term NIV [9, 10] and was then validated in patients receiving LTOT [16]. The SRI has seven subscales: Respiratory Complaints, Physical Functioning, Attendant Symptoms and Sleep, Social Relationships, Anxiety, Psychological Well-Being, and Social Functioning. The summary score is obtained from the summary of the seven subscales. In each questionnaire, the score ranges from 0 to 100; higher scores indicate a worse HRQL in the SGRQ and MRF, and vice versa, in the SRI.

Dyspnea during activities of daily living was evaluated by the modified Medical Research Council (mMRC) dyspnea scale [12, 17]. The mMRC is a unidimensional 5-point scale (0–4) based on degrees of various physical activities that precipitate dyspnea. Higher scores indicate worse dyspnea. Psychological status was assessed using the Japanese version of the Hospital Anxiety and Depression Scale (HADS) [12, 18], which measures anxiety and depression. Each subscale score ranges from 0 to 21, with higher scores indicating a poor psychological status.

### 2.3. Longitudinal Study

To compare the changes in the three HRQL measures (SGRQ, MRF-26, and SRI), we assessed the measures in 103 patients who were alive but did not undergo lung transplantation one year after the baseline assessment.

### 2.4. Statistical Analysis

Results are expressed as the mean ± standard deviation. Score distributions of HRQL were evaluated using histograms and Kolmogorov–Smirmov tests. Reliability was analysed based on internal consistency, which was calculated by Cronbach's *α* coefficients. Spearman rank correlation tests were performed to analyse the relationships between two sets of data. Stepwise multiple regression analyses were used to identify the variables that could best predict the HRQL scores, using factors significantly correlated with each HRQL score as explanatory variables. Comparisons in the changes in HRQL scores between baseline and one year later were performed using Wilcoxon signed rank tests. A *p* value less than 0.05 was considered statistically significant. All statistical analyses were performed using EZR (Saitama Medical Center, Jichi Medical University, Saitama, Japan), which is a graphical user interface for R (The R Foundation for Statistical Computing, Vienna, Austria). More precisely, it is a modified version of R commander designed to add the statistical functions frequently used in biostatistics [19].

## 3. Results

### 3.1. Patients

The baseline characteristics of 199 patients (102 men and 97 women) are shown in Table 1. In total, 175 patients (87.9%) were treated with LTOT and 11 (5.5%), with long-term NIV. The indications of lung transplant were as follows: interstitial pneumonia (*n* = 99, 49.7%), pulmonary complications of hematopoietic stem cell transplantation (*n* = 29, 14.6%), pulmonary hypertension (*n* = 17, 8.5%), LAM (*n* = 15, 7.5%), COPD (*n* = 13, 6.5%), bronchiectasis (*n* = 11, 5.5%), and others (*n* = 15, 7.5%).

### 3.2. Baseline HRQL Scores

**Figure 1:**
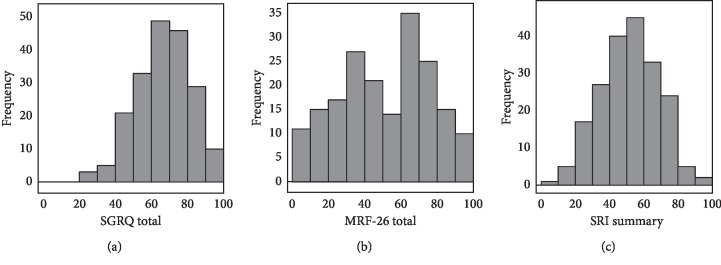
Frequency distribution of each HRQL score. (a) The SGRQ total; (b) the MRF-26 total; and (c) the SRI summary. Higher scores indicate worse HRQL in the SGRQ and MRF, and vice versa, in the SRI.

The frequency distribution histograms of Total and Summary scores of each HRQL measure are shown in Figure 1. The SGRQ total, MRF-26 total, and SRI summary appeared to show nearly normal distributions based on Kolmogorov–Smirmov tests (*p*=0.85, 0.11, and 0.66, respectively). Cronbach's *α* coefficients for Total and Summary of each HRQL measures were 0.84 (SGRQ), 0.90 (MRF-26), and 0.83 (SRI), indicating high reliability of internal consistency.

The baseline HRQL scores are presented in Table 2. The mean score of the SGRQ total was 66.4, leaning toward worse ends, as compared to the MRF-26 total (mean = 51.6) and the SRI summary (mean = 52.0). Especially, the mean score of the SGRQ Activities was high (83.0), and 29 patients (14.5%) presented the maximal score, showing the ceiling effect. In comparison, the mean score of all the MRF-26 domains and SRI subscales was around 50 (range, 44.4–59.3). However, with regard to the MRF-26 Daily activities, 14 (7.0%) and 16 patients (8.0%) presented the minimal and maximal scores, respectively, showing both floor and ceiling effects. The SRI seemed to present the least floor and ceiling effects among the three questionnaires.

### 3.3. Validity

Correlations between the SGRQ and the MRF-26 and SRI are shown in Table 3. The MRF-26 Total and SRI Summary were strongly correlated with the SGRQ total (Spearman's rank correlation coefficient (Rs) = 0.78 and −0.74, *p* < 0.001, respectively). All the domains of the MRF-26 and SRI were significantly correlated with the SGRQ (Rs = 0.19 to 0.78, *p* < 0.05). Except for the Total and Summary scores, strong relationships (Rs > 0.60, *p* < 0.001) were found between the SGRQ Activities, and the MRF-26 Daily activities and SRI Physical Functioning; and between the SGRQ Impacts and the MRF two domains, SRI Respiratory Complaints, Physical Functioning, Anxiety, and Social Functioning.

### 3.4. Relationship between HRQL and Clinical Measurements

SGRQ, MRF-26, and SRI measures were not significantly correlated with age, BMI, and blood parameters of albumin, hemoglobin, creatinine, and C-reactive protein (*p* > 0.05). They were not or only weakly significantly correlated with partial pressure of arterial oxygen (PaO_2_) and partial pressure of arterial carbon dioxide (PaCO_2_). While the SGRQ was weakly but significantly correlated with both FEV_1_ (%predicted) and FVC (%predicted) (Rs = −0.42 to −0.31, *p* < 0.05), the MRF-26 Perceived disability and some SRI subscales were correlated with neither FEV_1_ nor FVC (*p* > 0.05). They were weakly to moderately significantly correlated with 6MWD (Rs = 0.22 to 0.47, *p* < 0.05), except for insignificant relationships of the SRI Respiratory Complaints and Attendant Symptoms and Sleep.

With regard to the relations with dyspnea and psychological status, all the SGRQ, MRF-26, and SRI were significantly correlated with mMRC dyspnea (Rs = 0.30 to 0.67, *p* < 0.05), and anxiety (Rs = 0.29 to 0.66, *p* < 0.05) and depression (Rs = 0.26 to 0.67, *p* < 0.05) of the HADS. Except for the Total and Summary scores, the SGRQ Activities and Impacts, and SRI Physical Functioning were strongly correlated with mMRC dyspnea (Rs > 0.60, *p* < 0.001), while the SGRQ Impacts, MRF-26 Perceived disability, and SRI Psychological Well-Being were strongly correlated with anxiety or depression of the HADS (Rs > 0.60, *p* < 0.001) (see Table 4).

We then performed multiple regression analyses to investigate the factors predicting each of the HRQL Total and Summary scores. As observed in Table 5, 71%, 64%, and 64% of the variance in the SGRQ, MRF-26, and SRI, respectively, were explained in the present models. With regard to physiological measures, FVC significantly accounted for 7% and 5% of the variance in the SGRQ and MRF-26, respectively, while 6MWD accounted for 4%, 5%, and 5% of the variance in the SGRQ, MRF, and SRI, respectively. mMRC dyspnea and HADS anxiety and depression more strongly accounted for 12% to 28% of the variance in the SGRQ, MRF, and SRI.

### 3.5. Responsiveness

**Figure 2:**
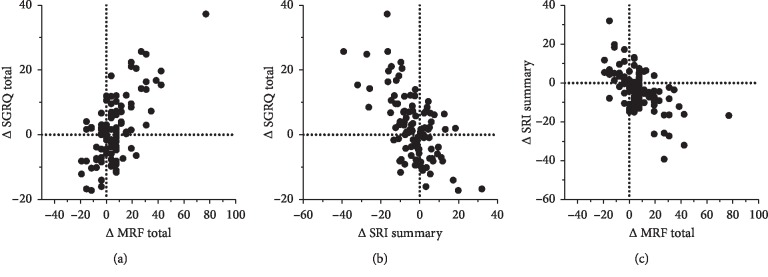
Scatter plots between the changes (Δ) in each HRQL measure. (a) The SGRQ total versus the MRF-26 total; (b) the SGRQ total versus the SRI summary; and (c) the SRI summary versus the MRF-26 total.

Of the 199 patients enrolled, 103 patients were included in the one-year study. The reasons for exclusion were as follows: 32 patients who underwent lung transplantation, 32 patients who died within 1 year, 26 patients who missed the 1-year evaluation, 5 patients who provided insufficient answers, and one patient who transferred to another hospital. To assess the responsiveness of each questionnaire, we compared the HRQL scores between baseline and at one year in 103 patients who had not undergone lung transplantation and who agreed to undergo a follow-up assessment. The MRF-26 Total and SRI Summary significantly worsened from 44.9 ± 24.3 to 52.1 ± 26.2 (*p* < 0.001) and from 55.0 ± 17.0 to 51.6 ± 18.0 (*p*=0.010), respectively, while the SGRQ total showed a marginally significant deterioration from 62.5 ± 15.3 to 65.1 ± 16.9 (*p*=0.040) (Table 6). Except for the Total and Summary scores, statistically striking worsening in the HRQL (*p* < 0.01) was found in the MRF-26 two domains and the SRI Respiratory Complaints, Anxiety, and Social Functioning, but not in the SGRQ. With regard to the interrelationships between the changes in the HRQL measures, the changes in the SGRQ total, MRF-26 total, and SRI summary had moderate significant relationships with each other (Rs = 0.54 to 0.56, *p* < 0.001) (Figure 2). Additionally, we compared the baseline and 1-year HRQL in the IP or non-IP group (*n* = 47 and 56, respectively). In the IP group, MRF-26 Total and SRI Summary indicated significant deterioration from 40.8 ± 22.4 to 51.7 ± 26.1 (*p* < 0.001) and 59.3 ± 15.5 to 54.0 ± 18.1 (*p*=0.004), respectively, while SGRQ did not show a statistically significant change from 60.6 ± 14.6 to 64.6 ± 17.6 (*p*=0.07). In the non-IP group, MRF-26 total showed a significant deterioration from 48.4 ± 25.3 to 52.4 ± 26.1 (*p*=0.009) but no significant changes were observed in SRI summary from 51.4 ± 17.5 to 49.6 ± 17.9 (*p*=0.06) and SGRQ from 64.1 ± 15.9 to 65.5 ± 16.5 (*p*=0.23). Thus, as summarized in Table 7, baseline HRQL in IP patients was better than baseline HRQL in non-IP patients, but that deterioration in HRQL in IP patients was greater than that in non-IP patients.

## 4. Discussion

The present study demonstrated that compared with the respiratory-specific SGRQ measures, CRF-specific HRQL measures of the MRF-26 and SRI were valid, discriminative, and responsive in patients waitlisted for lung transplantation. In addition, they were better than the SGRQ in terms of score distribution and responsiveness.

We anticipated that CRF-specific questionnaires would be more suitable than the widely used SGRQ in assessing HRQL in patients waitlisted for lung transplantation from the point of their underlying disease and its severity. Items on the MRF-26 and SRI are specific to patients with CRF, irrespective of the underlying diseases, who are usually treated with LTOT and/or NIV. Therefore, these patients would be close to lung transplant candidates. In contrast, the SGRQ was originally validated in mild to severe airway diseases such as COPD, and it was not specific to patients with CRF. This is confirmed by the fact that idiopathic pulmonary fibrosis (IPF)-specific SGRQ has been developed separately after revision of the original version because some SGRQ items have weak measurement properties in patients with IPF [20], who assumed a majority in the present population.

As a result, while the mean values were around the middle of the score range in the MRF-26 and SRI, they skewed toward worse ends in the SGRQ (Table 2). In addition, the ceiling effects were remarkable in the SGRQ Activities. This could be attributed to a mismatch between the items in the SGRQ and the disease severity or characteristics of the patients. In contrast, we consider that the higher tendency of the ceiling and floor effects in the MRF-26, as shown in Figure 1 and Table 2, would be attributed to its simple structure with “yes” or “no” responses for 26 items. While the MRF-26 has an advantage of ease of completion, the SRI seemed to be the most balanced among the three measures in terms of score distribution.

The present cross-sectional analysis of the relationships between three HRQL measures and between HRQL and clinical measures indicated that similar to the SGRQ, the MRF-26 and SRI reflected physiological and psychological impairments in patients waitlisted for lung transplantation. As expected, stronger correlations were observed in their relations with dyspnea and psychological status than with physiological measures of pulmonary function and 6MWD. This was confirmed through multiple regression analysis to predict the Total and Summary HRQL scores. Dyspnea, anxiety, and depression scores accounted for 54%–61% of the variance, while pulmonary function and exercise capacity accounted for only 5%–11% of the variance. This is consistent with a similar analysis of contributive factors of different HRQL measures in patients receiving long-term NIV [14] who severely deteriorated physiologically, as observed in lung transplant candidates.

Responsiveness of the MRF-26 and SRI was confirmed by comparing it against that of the SGRQ. Although their Total and Summary scores showed a statistically significant worsening, the significance was stronger in the MRF-26 and SRI than in the SGRQ. This might be because the mean score of the SGRQ leaned toward worse ends at baseline, and its responsiveness was limited owing to the ceiling effect, which was especially striking in the SGRQ Activities score from 79.9 (baseline) to 82.3 (1 year), as compared with the MRF and SRI. Moderate significant correlations between the changes in the three questionnaires (Figure 2) indicated that they reflected some degrees of the changes in HRQL in common and that the responsive feature of the MRF-26 and SRI would be justified. Patients with COPD who showed a deterioration in the HRQL during one year had a higher chance of an exacerbation, hospitalization, or death during the 2 years of follow-up [21]. Thus, although the baseline HRQL with MRF-26 and SRI had prognostic value in patients with CRF [12, 22], their changes would be associated with poor health outcomes. Reducing the waitlist mortality rate is an important aim in the Lung Allocation System (LAS) era. For this purpose, routine monitoring HRQL with such responsive measures may be useful in detecting patients at risk for premature death, although further studies are warranted.

With the improvement in survival rates after lung transplantation, the importance in patient-reported outcomes has recently increased [23]. After the implementation of the LAS in the United States, HRQL has been reported to improve [24]. However, unfortunately, the LAS itself comes from physiological measures and underlying diseases, and it may not adequately reflect the health and symptoms of the patients [25]. Hence, the importance of appropriate lung transplant-specific questionnaires cannot be undermined. These questionnaires have recently been developed [26–28], and future studies to assess their function and utility are expected.

This study includes some limitations. First, we have mainly focused on the situation before lung transplantation. Second, recently, the development of a lung transplant-specific instrument had been reported [29]. The present study, however, had been conducted before the novel questionnaire was developed. Third, we aimed to compare the responsiveness of the three different instruments rather than to examine real longitudinal changes in HRQL. In the latter case, to avoid underestimating the change in HRQL, we should not exclude the patients who dropped out due to death, as these tend to have a greater deterioration in HRQ or those who experienced exacerbations. We aim to calculate the “real” change in HRQL using another statistical methodology in the future.

## 5. Conclusions

In conclusion, CRF-specific HRQL measures of the MRF-26 and SRI are valid and useful in patients waitlisted for lung transplantation. They may reflect health impairments of patients more appropriately than the currently used HRQL measures do. In the future, we aim to assess their function as risk stratification measures irrespective of underlying diseases in order to reduce the waitlist mortality rate more than this.

## Figures and Tables

**Table 1:** Baseline characteristics of 199 patients.

	Mean ± SD
Age, years	45.4 ± 11.1
Sex, male/female	102/97
BMI, kg/m^2^	19.6 ± 3.88
PaO_2_, mmHg	79.3 ± 26.2
PaCO_2_, mmHg	46.2 ± 10.1
%FEV_1_, %predicted	42.1 ± 19.8
%FVC, %predicted	50.9 ± 21.2
6MWD, m	302 ± 144
mMRC dyspnea (0–4)	2.74 ± 1.14
HADS anxiety (0–21)	5.47 ± 4.47
HADS depression (0–21)	6.82 ± 4.47

BMI: body mass index; PaO_2_: partial pressure of arterial oxygen; PaCO_2_: partial pressure of arterial carbon dioxide; FEV_1_: forced expiratory volume in one second; FVC: forced vital capacity; 6MWD: 6-minute walk distance; mMRC: modified Medical Research Council; HADS: Hospital Anxiety and Depression Scale.

**Table 2:** Baseline scores of three HRQL questionnaires in 199 waitlisted patients for lung transplantation.

	Items	Mean ± SD	Minimal score, *N* (%)^a^	Maximal score, *N* (%)^a^
SGRQ				
Symptoms	8	67.4 ± 21.4	0 (0.0%)	3 (1.5%)
Activities	16	83.0 ± 15.8	0 (0.0%)	29 (14.5%)
Impacts	26	56.9 ± 19.5	0 (0.0%)	1 (0.5%)
Total	50	66.4 ± 15.5	0 (0.0%)	0 (0.0%)

MRF-26				
Daily activities	13	52.0 ± 30.9	14 (7.0%)	16 (8.0%)
Perceived disability	13	54.1 ± 24.7	4 (2.0%)	3 (1.5%)
Total	26	51.6 ± 25.3	2 (1.0%)	2 (1.0%)

SRI				
Respiratory complaints	8	52.8 ± 22.8	0 (0.0%)	1 (0.5%)
Physical functioning	6	44.4 ± 23.2	3 (1.5%)	0 (0.0%)
Attendant symptoms and sleep	7	59.3 ± 22.7	1 (0.5%)	2 (1.0%)
Social relationships	6	49.3 ± 22.8	2 (1.0%)	0 (0.0%)
Anxiety	5	49.8 ± 23.1	3 (1.5%)	1 (0.5%)
Psychological well-being	9	54.0 ± 20.3	1 (0.5%)	0 (0.0%)
Social functioning	8	54.5 ± 21.1	0 (0.0%)	2 (1.0%)
Summary	49	52.0 ± 17.9	0 (0.0%)	0 (0.0%)

All theoretical score ranges are 0–100 on each measurement. For the SGRQ and MRF-26, maximum scores indicate poor health status, and for the SRI, minimum scores indicate poor health status. ^a^Number of patients. SGRQ: St. George's Respiratory Questionnaire; MRF: Maugeri Respiratory Failure Questionnaire; SRI: Severe Respiratory Insufficiency Questionnaire.

**Table 3:** Relations of the MRF-26 and SRI with the SGRQ.

	SGRQ			
	Symptoms	Activities	Impacts	Total
MRF-26				
Daily activities	0.38	0.70	0.67	0.72
Perceived disability	0.41	0.46	0.67	0.66
Total	0.43	0.67	0.75	0.78

SRI				
Respiratory complaints	−0.57	−0.54	−0.64	−0.68
Physical functioning	−0.29	−0.60	−0.68	−0.68
Attendant symptoms and sleep	−0.36	−0.32	−0.48	−0.47
Social relationships	−0.21	−0.33	−0.49	−0.47
Anxiety	−0.37	−0.51	−0.65	−0.64
Psychological well-being	−0.19	−0.29	−0.55	−0.49
Social functioning	−0.27	−0.51	−0.63	−0.62
Summary	−0.41	−0.57	−0.75	−0.74

All values indicate Spearman's rank correlation coefficients (*p* < 0.05). SGRQ: St. George's Respiratory Questionnaire; MRF: Maugeri Respiratory Failure Questionnaire; SRI: Severe Respiratory Insufficiency Questionnaire.

**Table 4:** Relationship between HRQL and clinical measurements.

	SGRQ				MRF-26			SRI							
	Sym	Act	Imp	Total	Dai	Per	Total	RC	PF	AS	SR	AX	WB	SF	Sum
Age	—	—	—	—	—	—	—	—	—	—	—	—	—	—	—
BMI	—	—	—	—	—	—	—	—	—	—	—	—	—	—	—
Albumin	—	—	—	—	—	—	—	—	—	—	—	—	—	—	—
Hemoglobin	—	—	—	—	—	—	—	—	—	—	—	—	—	—	—
Creatinine	—	—	—	—	—	—	—	—	—	—	—	—	—	—	—
CRP	—	—	—	—	—	—	—	—	—	—	—	—	—	—	—
PaO_2_	—	—	—	—	—	—	—	—	0.27	—	—	—	—	—	—
PaCO_2_	0.22	—	—	0.25	0.29	—	0.24	−0.32	—	—	—	—	—	—	—
%FEV_1_	−0.33	−0.42	−0.33	−0.41	−0.41	—	−0.34	0.30	0.37	—	0.24	0.27	—	0.30	0.34
%FVC	−0.31	−0.42	−0.38	−0.42	−0.41	—	−0.41	0.40	0.33	—	—	—	—	—	0.31
6MWD	−0.27	−0.47	0.41	−0.47	−0.39	−0.27	−0.37	—	0.41	—	0.26	0.25	0.22	0.40	0.35
mMRC dyspnea	0.30	0.64	0.62	0.67	0.55	0.41	0.55	−0.36	−0.63	−0.31	−0.38	−0.44	−0.31	−0.53	−0.54
HADS anxiety	0.29	0.40	0.65	0.60	0.46	0.63	0.59	−0.43	−0.49	−0.48	−0.57	−0.57	−0.60	−0.53	−0.66
HADS depression	0.26	0.40	0.67	0.61	0.53	0.58	0.61	−0.40	−0.54	−0.36	−0.54	−0.54	−0.59	−0.55	−0.62

Missing values (—) indicate no significant relationships (*p* > 0.05). SGRQ: St. George's Respiratory Questionnaire; Sym: symptoms; Act: activities; Imp: impacts; MRF: Maugeri Respiratory Failure Questionnaire; Dai: daily activities; Per: perceived disability; SRI: Severe Respiratory Insufficiency Questionnaire; RC: respiratory complaints; PF: physical functioning; AS: attendant symptoms and sleep; SR: social relationships; AX: anxiety; WB: psychological well-being; SF: social functioning; Sum: summary; BMI: body mass index; CRP: C-reactive protein; PaO_2_: partial pressure of arterial oxygen; PaCO_2_: partial pressure of arterial carbon dioxide; FEV_1_: forced expiratory volume in one second; FVC: forced vital capacity; 6MWD: 6-minute walk distance; mMRC: modified Medical Research Council; HADS: hospital anxiety and depression scale.

**Table 5:** Stepwise multiple regression analyses to predict the SGRQ, MRF-26 and SRI scores.

	SGRQ total	MRF-26 total	SRI summary
PaCO_2_	—	—	—
%FEV_1_	—	—	—
%FVC	0.07	0.05	—
6MWD	0.04	0.05	0.05
mMRC dyspnea	0.28	0.21	0.17
HADS anxiety	0.12	0.14	0.25
HADS depression	0.21	0.19	0.17
Cumulative *R*^2^	0.71	0.64	0.64

All values represent coefficient of determination (*R*^2^). Missing values (—) indicate no significant relationships. SGRQ: St. George's Respiratory Questionnaire; MRF: Maugeri Respiratory Failure Questionnaire; SRI: Severe Respiratory Insufficiency Questionnaire; PaCO_2_,:partial pressure of arterial carbon dioxide; FEV_1_: forced expiratory volume in one second; FVC: forced vital capacity; 6MWD: 6-minute walk distance; mMRC: modified Medical Research Council; HADS: Hospital Anxiety and Depression Scale.

**Table 6:** Comparison of HRQL scores between baseline and 1 year in 103 patients.

	Baseline	1 year	*p* value
SGRQ			
Symptoms	63.5 ± 20.3	64.5 ± 20.8	0.33
Activities	79.9 ± 14.8	82.3 ± 15.5	0.019
Impacts	52.3 ± 18.9	55.4 ± 20.7	0.062
Total	62.5 ± 15.3	65.1 ± 16.9	0.040

MRF-26			
Daily activities	44.2 ± 30.0	52.8 ± 33.0	<0.001
Perceived disability	45.6 ± 23.9	51.4 ± 24.5	<0.001
Total	44.9 ± 24.3	52.1 ± 26.2	<0.001

SRI			
Respiratory complaints	57.0 ± 22.6	52.8 ± 22.9	<0.001
Physical functioning	48.9 ± 22.0	44.7 ± 22.2	0.017
Attendant symptoms and sleep	62.2 ± 23.2	59.8 ± 21.8	0.14
Social relationships	55.2 ± 18.3	53.0 ± 18.5	0.13
Anxiety	54.8 ± 22.0	49.5 ± 24.3	0.003
Psychological well-being	54.0 ± 18.5	53.8 ± 19.4	0.99
Social functioning	52.6 ± 22.6	47.8 ± 24.2	<0.001
Summary	55.0 ± 17.0	51.6 ± 18.0	0.010

Data are shown as mean ± SD. SGRQ: St. George's Respiratory Questionnaire; MRF: Maugeri Respiratory Failure Questionnaire; SRI: Severe Respiratory Insufficiency Questionnaire.

**Table 7:** Comparison of baseline and 1-year HRQL scores between IP and non-IP patients.

		Baseline	1 year	%change	*p* value
SGRQ total	IP	60.6 ± 14.6	64.6 ± 17.6	6.6	0.07
	Non-IP	64.1 ± 15.9	65.5 ± 16.5	2.2	0.23
MRF-26 total	IP	40.8 ± 22.4	51.7 ± 26.1	26.7	<0.001
	Non-IP	48.4 ± 25.3	52.4 ± 26.1	8.3	0.009
SRI summary	IP	59.3 ± 15.5	54.0 ± 18.1	8.9	0.004
	Non-IP	51.4 ± 17.5	49.6 ± 17.9	3.5	0.06

Data are shown as mean ± SD. HRQL: health-related quality of life; IP: interstitial pneumonia; SGRQ: St. George's Respiratory Questionnaire; MRF, Maugeri Respiratory Failure Questionnaire; SRI: Severe Respiratory Insufficiency Questionnaire.

## Data Availability

The data used to support the findings of this study are available from the corresponding author upon reasonable request.

## Abbreviations

HRQL: Health-related quality of life
SGRQ: St. George's Respiratory Questionnaire
CRF: Chronic respiratory failure
MRF: Maugeri Respiratory Failure Questionnaire
SRI: Severe Respiratory Insufficiency Questionnaire
SF-36: Medical Outcomes Study 36-item short form
COPD: Chronic obstructive pulmonary disease
NIV: Noninvasive ventilation
LTOT: Long-term oxygen therapy
BMI: Body mass index
FEV_1_: Forced expiratory volume in one second
FVC: Forced vital capacity
6MWD: 6-minute walk distance
LAM: Lymphangioleiomyomatosis
mMRC: Modified Medical Research Council
HADS: Hospital Anxiety and Depression Scale
PaO_2_: Partial pressure of arterial oxygen
PaCO_2_: Partial pressure of arterial carbon dioxide
LAS: Lung allocation system.
